# Qualitative and quantitative retrieval analysis of a ball-and-socket cervical disc replacement

**DOI:** 10.1016/j.xnsj.2025.100768

**Published:** 2025-07-05

**Authors:** Jenna M. Wahbeh, Sophia N. Sangiorgio, Sang-Hyun Park, G. Bryan Cornwall, Neha V. Kulkarni, Roberto Chiesa, Edward Ebramzadeh

**Affiliations:** a The J. Vernon Luck, Sr., M.D. Orthopaedic Research Center, The Luskin Orthopaedic Institute for Children in Alliance with UCLA, Los Angeles, CA, United States; b Henry Samueli School of Engineering Department of Bioengineering, University of California, Los Angeles, CA, United States; c David Geffen School of Medicine Department of Orthopaedic Surgery, University of California, Los Angeles, CA, United States; d Shiley-Marcos School of Engineering, University of San Diego, San Diego, CA, United States; e Department of Chemistry, Materials, and Chemical Engineering, Politecnico Milano, Milan, Italy

**Keywords:** Cervical disc replacement, Cervical arthroplasty, PCM device, Device fixation, Retrieval analysis, Migration, Device design, Initial stability

## Abstract

**Background:**

The Porous Coated Motion (PCM) is a ball-and-socket cervical disc replacement with excellent reported short-term clinical outcome. However, longer-term studies identified migration as a common cause of implant removal and the device was withdrawn from the market. Given these discrepancies, retrieval analyses are crucial to assess whether preclinical testing accurately predicts clinical performance. This study aimed to quantitatively and qualitatively analyze retrieved PCM devices to identify primary reasons for removal and assess the impact of observed damage on overall device fixation.

**Methods:**

Thirty-seven PCM devices were received for postmarket surveillance. Nondestructive analysis included visual examination, photographic documentation, and radiographic review. Analytical measurements were performed using a coordinate measuring machine to assess articulating surfaces or a digital microscope for endplate surface feature characterization. Oxidation analysis was performed on all devices with adequate handling and storage conditions, and histopathology was performed when tissue samples were available.

**Results:**

Twenty-five devices met the inclusion criteria for this study. The mean patient age at retrieval was 45.3±13.5 years, with an average time-in-vivo of 121±15.6 days. Anterior migration was the most common reason for removal, reported in 17 cases, with the inferior convex polyethylene component predominately migrating. Additionally, 17 devices had a focalized deviation on the posterior quadrant of the articulating polyethylene ball, 11 of which had evidence of radiographic clinical migration. Histopathology and metrology findings indicated that wear debris did not contribute to clinical failure.

**Conclusions:**

The findings of the present study, specifically the minimal bony ongrowth, lack of extraction damage, and radiographic imaging, indicated that most devices were removed due to migration. Metrology analysis revealed a depression on the posterior edge of the inferior endplate polyethylene ball, which correlated with anterior slippage. This may be a distinctive feature of the PCM’s relatively large ball-and-socket design that led to increased stress during extension, causing anterior migration.

## Introduction

The Porous Coated Motion (PCM) cervical disc replacement (NuVasive, San Diego, CA), introduced in 2002, which received FDA approval in 2012, had been reported to have excellent early clinical outcome [1,2]. Specifically, prospective, randomized clinical trials concluded that the PCM had favorable outcome with few reports of serious adverse events or secondary surgeries and high patient-reported outcome measures, in comparison to the current standard of treatment, anterior cervical discectomy and fusion [[3], [4], [5], [6]]. In contrast, recent reviews of the Manufacturer and User-Facility Device Experience database cervical disc replacement (CDR) adverse events have revealed that the PCM experienced a high number of migration-related complications [7,8].

While the total incidence of device failure due to migration is unknown, the PCM was withdrawn from the market and is no longer commercially available. However, many of the PCM design principles are shared by current models of CDRs still on the market [9]. The PCM is a cobalt-chromium molybdenum alloy metal on ultrahigh molecular weight polyethylene ball-and-socket device with a titanium (Ti) and calcium phosphate porous coating on its outer surfaces and three rows of “V-Teeth” on the superior and inferior endplates [[10], [11], [12], [13]]. Many currently available cervical disc replacements incorporate similar design elements, such as fins, teeth, or keels, and surface treatments to promote initial fixation and long-term bony ongrowth, or a ball-and-socket articulation type for unconstrained motions [[13], [14], [15], [16], [17]].

Retrieval analysis allows for a comprehensive evaluation of cervical device clinical performance by considering relevant patient, surgeon, and implant-related factors, holistically [18]. Given the inconsistencies between clinical literature and adverse event reports surrounding the prevalence of migration, retrieval analyses are crucial for determining if preclinical testing is adequately predicting clinical performance and inform decisions on future preclinical and clinical studies. Therefore, the goal of the present study was to analyze a cohort of retrieved PCM cervical devices for the reported reasons of failure and any quantitative or qualitative assessments of damage. Specifically, this study focused on the success of the relevant mechanical design choices on this device and their contribution, or lack of, to overall fixation.

## Methods

Thirty-seven PCM devices were received for postmarket surveillance at our independent academic research center between 2013 and 2015. Each implant retrieval had a corresponding deidentified clinical record with various amounts of relevant information such as demographics, reasons for removal, and time in vivo. Each implant was analyzed using nondestructive approaches, including visual examination, photographic documentation, and analytical measurements. For inclusion in the current study, devices needed to possess a minimum amount of clinical data and present “V-teeth” on the outer endplates, a feature of the newest generation of the PCM.

### Clinical information

Deidentified demographic information was available for most devices, including sex and age of the patient, size of the device, the cervical level of operation, dates of implantation and removal, and adjacent fusions or devices, if any. The demographic information was compiled into a table, and noted when information was not provided. Provided clinical records indicated that surgeon-stated reasons for removal were primarily migration and improper implant sizing.

### Nondestructive visual analysis

Following decontamination of all components, the gross condition of the device was visually assessed for evidence of in vivo damage, such as scratching, polishing, and mechanical damage. Damage attributed to extraction, as well as the extent and location of bony ongrowth, was documented in each case. Detailed overview photographs were taken to document these features.

Microscopic analysis was conducted on all explanted devices using a high-resolution digital microscope (Keyence VHX, Keyence). Photographic documentation focused on four surfaces of the device: the superior endplate footprint, the superior articulating surface, the inferior endplate footprint, and the polyethylene bearing surface. The articulating polyethylene liner surfaces (superior articulating surface and inferior polyethylene liner) were visually analyzed for any evidence of in vivo damage, including discoloration, scratches, pitting, or deformity. Higher magnifications were used to identify embedded particles and loss of machining lines in the polyethylene coating, as well as to identify more fine in vivo damage. Images of the superior and inferior endplate footprints focused on observations of extraction damage and bony ongrowth. Radiographic imaging, when available, was used to confirm implant sizing, migration, and reported reasons of removal.

### Analytical measurements

Analytical measurements were taken using a coordinate measuring machine on the polyethylene articulation surface for all received implants. The polyethylene surface was divided into 4 quadrants: anterior, lateral left, lateral right, and posterior. Measurements from the coordinate measuring machine were utilized to locate the maximum and minimum deviation on the polyethylene articulating surface. Deviations were also measured and compared to the allowable manufacturing tolerance.

Increased magnification with a high-resolution digital microscope (Keyence VHX, Keyence) was used to obtain measurements of surface roughness and device dimensions. Specifically, surface roughness of the superior and inferior endplate, maximum height of the superior and inferior endplate, and height of the V-teeth (measured from the surface of the footprint to the highest point on the teeth) were measured.

### Oxidation analysis

Oxidation analysis was performed on all specimens received and stored within 1 month of removal surgery, *N*=8 eight inferior polyethylene components, using microcomputed topography (micro-CT), small punch testing, and Fourier transformed infrared (FTIR) spectroscopy to analyze cause of cracks in the device. High-resolution micro-CT images were taken of 11.7 µm sections of the polyethylene components to examine subsurface cracks from oxidation and internal cracks from overloading and mechanical stresses. Small punch testing was performed on each polyethylene component in accordance with ASTM standard F2977-13.

Lastly, FTIR spectroscopy was performed to measure the intensity of carbonyl absorptions according to ASTM standard F2102-13 with a Nicolet 6700 FTIR Spectrophotometer (Thermo Fisher Scientific, Inc.). The oxidation index was calculated by dividing the oxidation peak by the normalization peak. Data was collected at the polyethylene articular surface inferior, middle, and superior sections along a top-to-bottom central line in the polyethylene components.

### Histological assessment

When available, histology was performed on soft tissue samples to examine the overall tissue features, identify the presence and extent of inflammatory cells, and determine whether wear debris may have been a contributing factor to failure.

### Statistical analysis

The statistical analyses were performed using SPSS version 19.0 (IBM Inc., Armonk, NY). Correlations between each of the continuous and ordinal variables were evaluated using Spearman’s correlation coefficient. For continuous variables, independent sample comparison tests (Student’s *t* test or one-way ANOVA) were used to assess the certainty in the observed differences between the means of two or more groups.

## Results

Thirty-seven devices were received at the Explant Laboratory. Of these devices, nine did not include any clinical data, and three were of an older generation device design with a different endplate design, and thus were excluded from the present study. Therefore, 25 devices met the inclusion criteria for the present retrieval study and were analyzed for failure mechanisms.

### Clinical information

The average patient age was 45.3±13.5 years. The average time in vivo was 121±115.6 days. The dates of provided prerevision images were compared to surgery dates, and it was determined that only two devices had prerevision images, noting migration taken more than 3 days prior to removal. In both cases, the prerevision radiograph was approximately 1 month prior to revision, changing the time of migration onset from 49 to 15 days and 257 to 220 days. This made no substantial difference in the overall time to removal; therefore, for consistency, all analyses were conducted using time in vivo. Of the 25 available devices and associated demographic information, the sexes of 10 patients were known, with 8 being female and 2 being male. There were 7 small implants, 13 medium implants, and 5 large implants. The majority of devices were implanted at C5-C6 (Table 1). Three devices had an adjacent fusion, 12 had no adjacent fusion, and 10 were unknown. Six devices had an adjacent PCM CDR, 8 had no adjacent device, and 11 were unknown. Time to revision was not significantly influenced by any clinical information (p>.10).

**Table 1:** Clinical information.

Average age	45.3±13.5 y
Average time in vivo	121±115.6 d
Gender	*Number of devices*
Male	2
Female	8
Unknown	15
Device size	
Small	7
Medium	13
Large	5
Cervical level	
C3–C4	1
C4–C5	3
C5–C6	8
C6–C7	5
Unknown	8

**Fig. 1:**
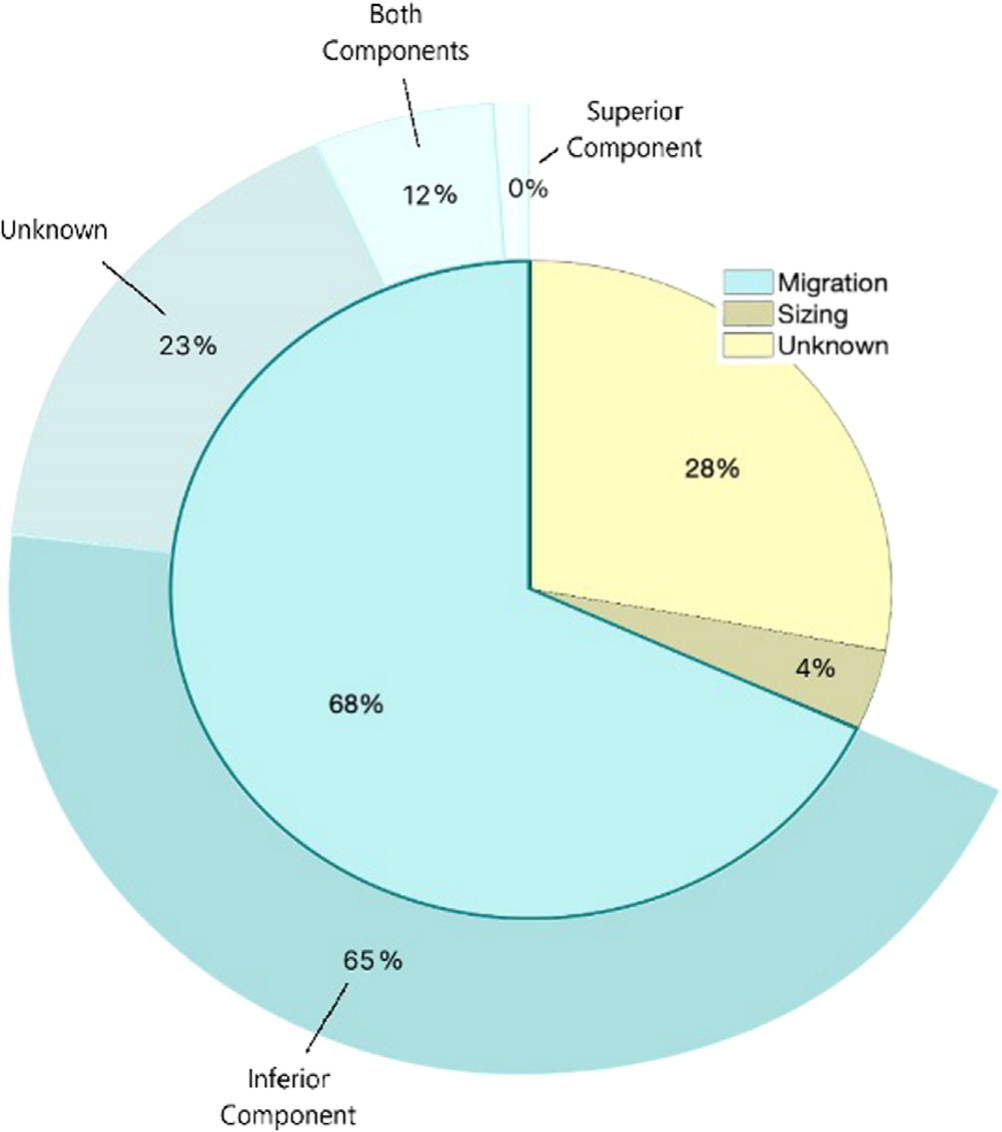
Surgical reason for removal, with specific migrating components depicted in the outer circle.

Device migration was the most commonly reported reason of removal (*n*=17), while one device was removed due to incorrect sizing and seven devices had unknown reasons for removal (Fig. 1). The average time in vivo for devices that were removed due to migration was 128.4±119.7 day, compared to the remaining devices removed after 69.5±78.5 days (p=.35). It is important to note that the device removed due to incorrect sizing was removed after only 14 days.

### Nondestructive visual analysis

**Fig. 2:**
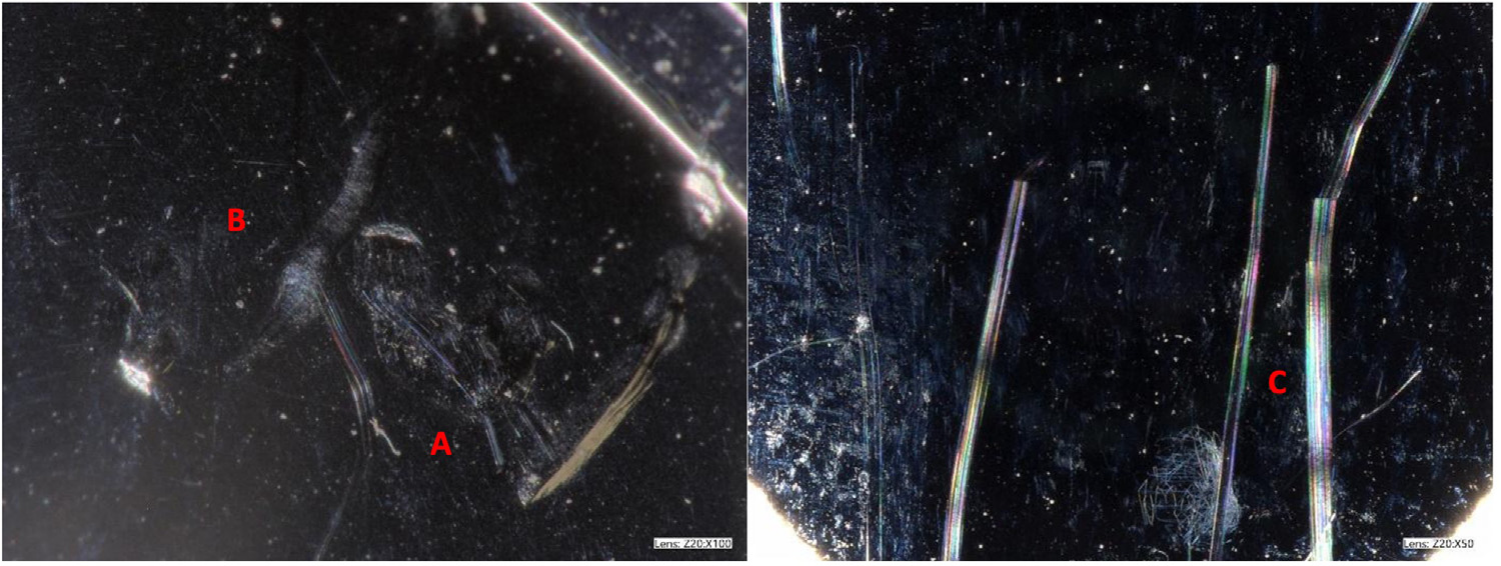
Representative image of visual observations of (A) indents, (B) fine scratches, and (C) deep scratches on the CoCrMo alloy articular surface of the superior endplate.

Nondestructive visual analysis was conducted on all components under high-resolution light microscopy. Light scratches were commonly observed, specifically in 21 cases, while deep scratches were relatively rare in only 3 cases. Indentations and third body damage were also infrequent observations (Fig. 2, Table 2).

**Table 2:** Visual analysis of retrieved components.

	Number of devices
Superior metal articular surface	
Light scratches	21
Deep scratches	3
Indents	9
Third body damage	4
Inferior UHMWPE articular surface	
Polishing/machining line loss	22
Discoloration	1
Location of UHMWPE polishing	
Focal	3
Peripheral	2
Both	17
Superior endplate fixation surface	
Removal damage	8
Bone ongrowth	6
Inferior endplate fixation surface	
Removal damage	9
Bone ongrowth	7

**Fig. 3:**
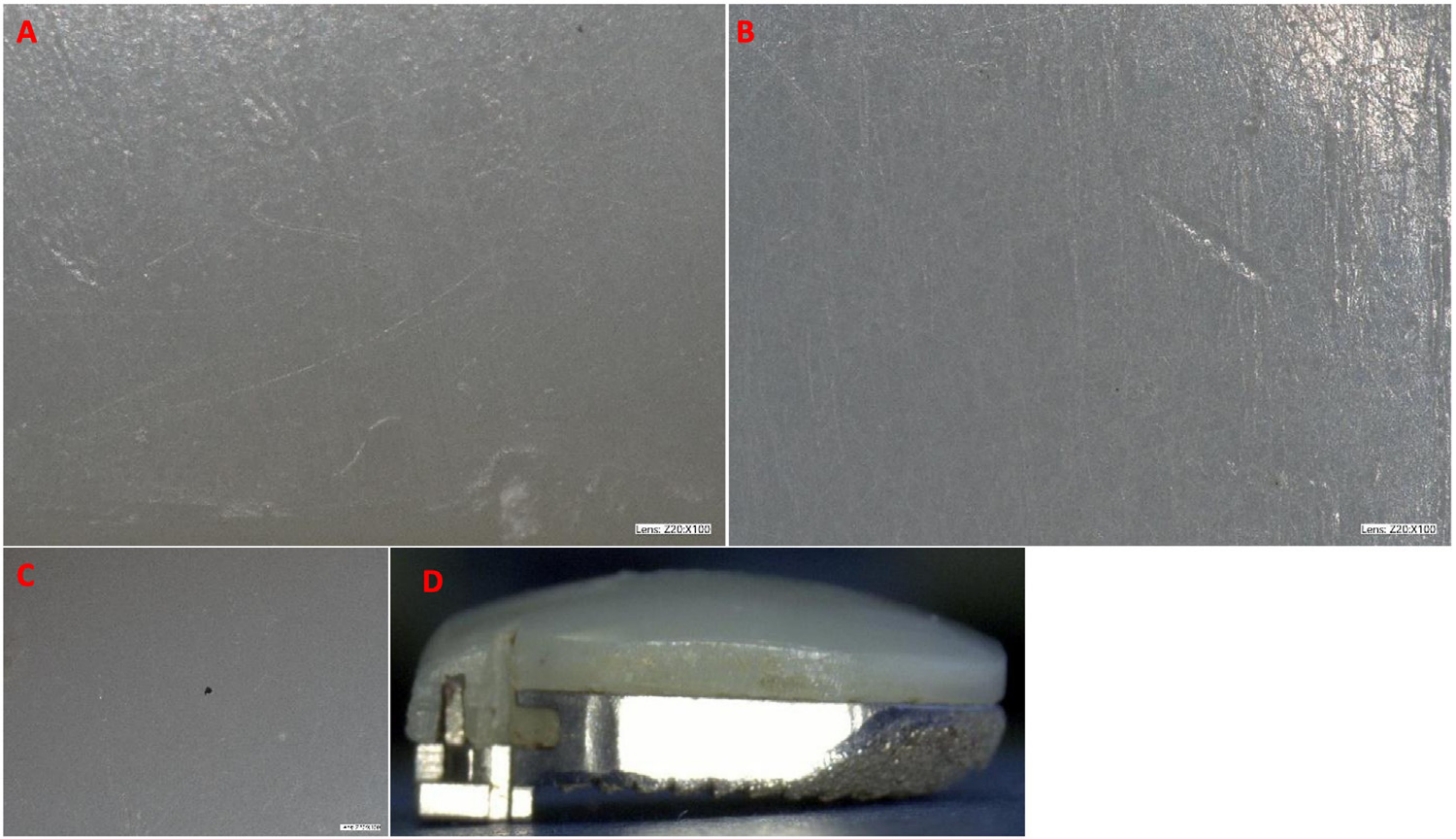
Representative images of visual analysis on UHMWPE of (A) fine scratches, (B) indentations, (C) loss of machining lines, and (D) UHMWPE discoloration.

On the articulating polyethylene surface, polishing was evident in 22 cases and discoloration in one case (Fig. 3). Loss of the residual tool marks from machining and wear polishing were observed in 22 cases. This was observed focally in 3 devices, peripherally in 2 devices, and both focally and peripherally in 17 devices (Table 2). In three cases, the residual tool marks from machining on the polyethylene bearing surfaces were completely intact,with no evidence of polishing.

Machining line loss/polishing was correlated with time in vivo in all quadrants (the anterior quadrant (p<.05), the left quadrant (p<.05), the right quadrant (p<.1), and the posterior quadrant (p<.05). However, there was no correlation between recorded wear and time to failure.

Regarding fixation, extraction damage was observed on 9 inferior and 8 superior endplates. Seven inferior endplates had evidence of bony ongrowth, while six superior endplates had evidence of bony ongrowth. Of these, only two inferior and superior endplates with bony ongrowth had corresponding removal damage (Table 2).

#### Radiographic analysis

**Fig. 4:**
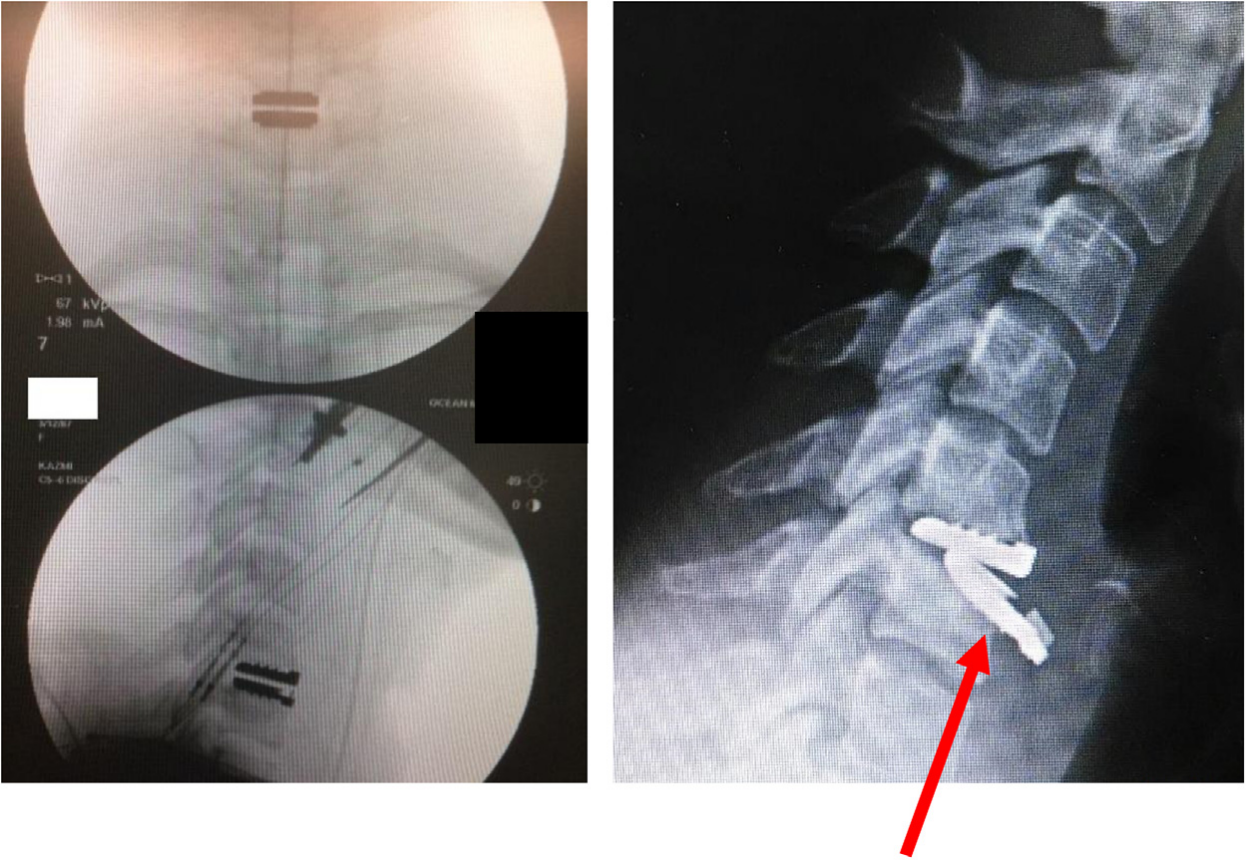
(Left) Intraoperative and (Right) preoperative radiographs with the arrow indicating anterior migration of the inferior component with the convex UHMWPE surface.

Seventeen devices were confirmed to have migrated prior to revision. Of the devices removed due to migration, the inferior component migrated in 11 of these cases, while migration of both the superior and inferior endplates was observed in just two cases (Fig. 4). No devices were reported to have only the superior component migrate. Four devices had an unknown observed migration component (Fig. 1). Additionally, all devices migrated anteriorly, and none migrated posteriorly. Subsidence was observed in one case in which the superior component subsided anteriorly by 30°, and the inferior component migrated anteriorly 25°.

### Analytical measurements

**Fig. 5:**
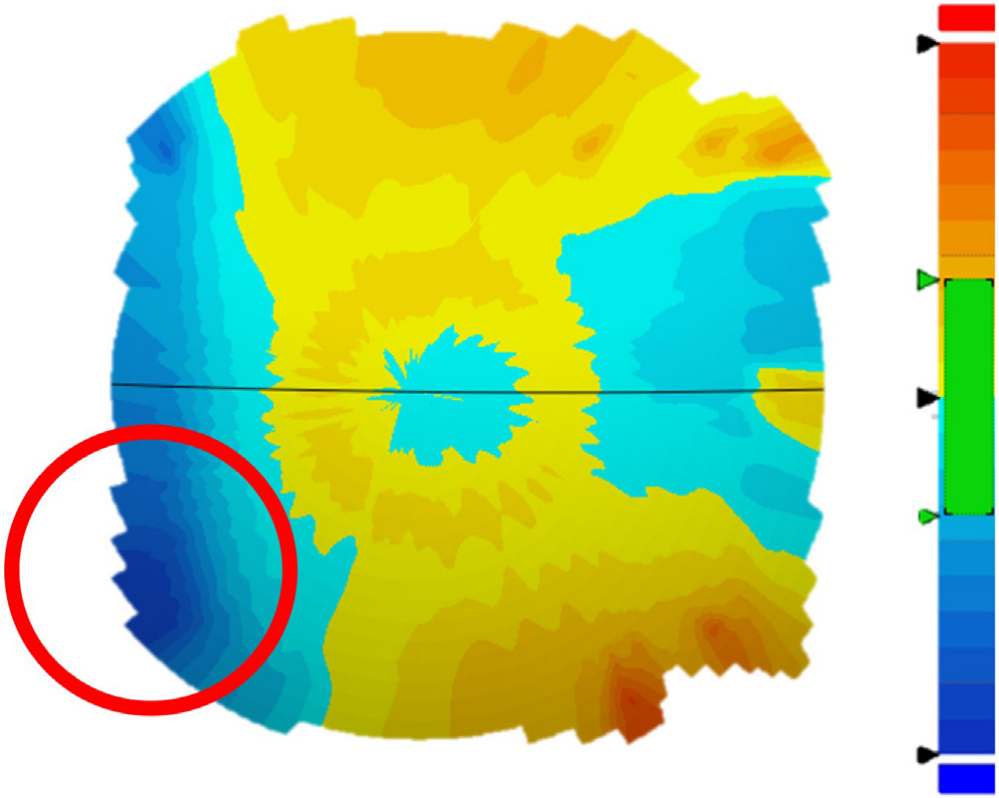
Metrology results indicating the focalized deviation the lateral right posterior edge, shown in the red circle.

The maximum deviation of the polyethylene bearing surface ranged from −212.2 to −8.4 µm with a mean of −53.1±48 µm. The severity of maximum deviations was not significantly correlated with time in vivo (p=.45, *ρ*=0.03). The areas of maximum deviation on the polyethylene bearing surface were typically localized in the posterior and anterior quadrants (Table 3). More specifically, according to metrology results, in 17 devices, a focalized deviation was specifically observed in a corner of the posterior quadrant toward the lateral left quadrant, 11 of which were removed due to anterior migration (Fig. 5). The average time in vivo for devices with a posterior, left deviation was 152±141.7 days, whereas without the deviation was 85.7±56.1 days; however, the difference was not statistically significant (p=.21). Eighteen individual polyethylene components had deviations at or below 30 µm, the manufacturer provided tolerance for radial deviation. For the remaining 7 devices, deviation was higher than 60 µm in only 5 cases (Table 3).

**Table 3:** UHMWPE liner CMM measurements.

	Number of devices
Location of maximum deviation polishing	
Posterior quadrant	12
Anterior quadrant	9
Left quadrant	1
Right quadrant	3
Average maximum deviation	
Less than 30 µm	18
Between 30 and 60 µm	2
More than 60 µm	5

**Fig. 6:**
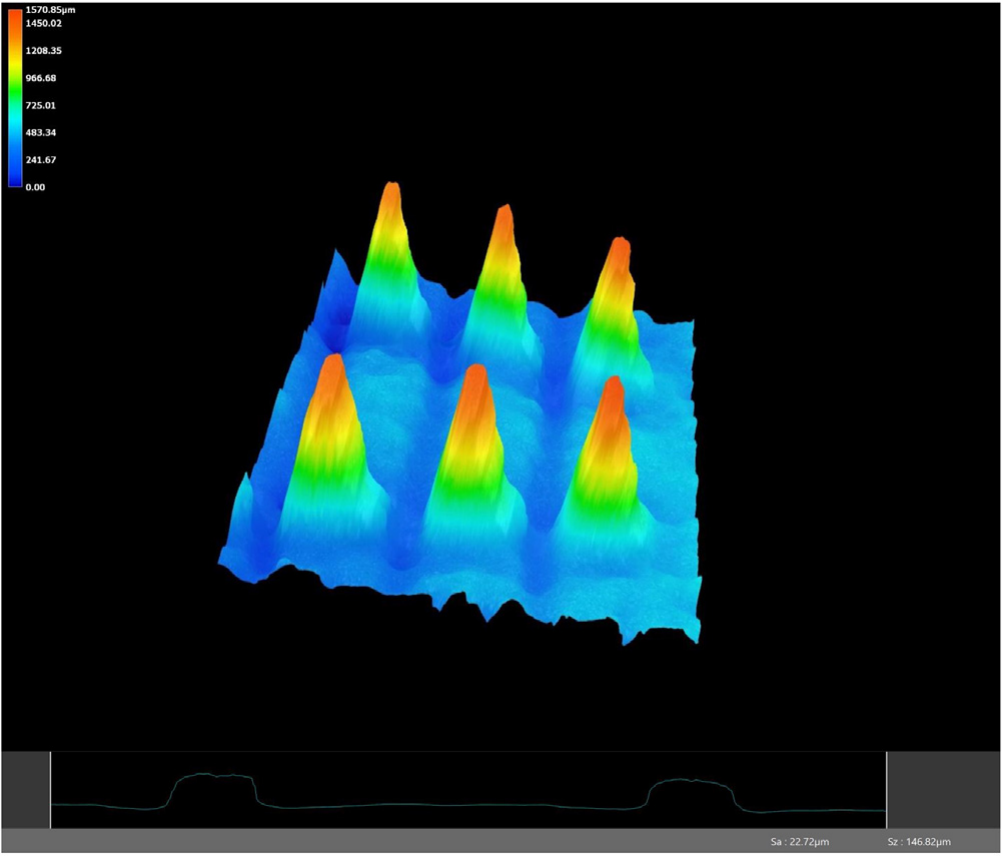
Representative image of the 3D endplate mapping in high-resolution microscopy analysis and a sample (bottom) surface roughness measurement indicating retention of original surface architecture and roughness.

Each device was examined under a high-resolution microscope, and 3D images were taken to assess the geometry of the device (Fig. 6). The average height of the V-teeth was 1.0±0.12 mm, compared to an as-manufactured device with measurements of 1.05 mm. The average surface roughness of the retrieved devices was 23.9±6.6 µm compared to an as-manufactured device with a roughness of 34.6 µm. The V-teeth height and surface roughness of an unused device were similar to the retrieved devices (p>.1, Table 4).

**Table 4:** Analytical measurements of new and retrieved devices from microscopy measurements.

	Average of retrievals	New, unused
V-teeth height	1.07±0.12 mm	1.05 mm
Superior endplate	1.12±0.06 mm	1.03 mm
Inferior endplate	1.02±0.08 mm	1.07 mm
Surface roughness	23.9±6.6 µm	34.6 µm
Superior endplate	22.1±5.4 µm	35.1 µm
Inferior endplate	25.8±7.6 µm	34.1 µm

Additionally, averages of V-teeth height and surface roughness were compared among devices with reported migration and with incorrect sizing or unknown causes of revision. The measured features were largely similar between devices with reported migration and devices without reported migration (p>.1, Table 5).

**Table 5:** Analytical measurements of devices with and without reported migration.

	Reported migration	No reported migration
Anchor height	1.08±0.08 mm	1.06±0.1 mm
Superior endplate	1.13±0.04 mm	1.10±0.07 mm
Inferior endplate	1.02±0.06 mm	1.03±0.1 mm
Surface roughness	24.0±5.5 µm	23.9±7.9 µm
Superior endplate	22.9±5.2 µm	21.1±5.3 µm
Inferior endplate	25.1±5.7 µm	26.7±9.2 µm

### Oxidation analysis

Interior cracks were identified in three of the eight specimens examined. One specimen exhibited cracks on the right lateral edge, suggesting that edge loading resulted in the crack. The other two specimens exhibited cracks both posteriorly and anteriorly from lateral right to lateral left. Analysis of micro-CT imaging suggested that most of these cracks were a result of oxidative damage, with the exception of one anterior crack, which was attributed to mechanical damage. Small punch testing found two polyethylene components in good condition or relatively undamaged. Six of the polyethylene components showed significant tapering because of oxidation, weakening the polyethylene structure. The FTIR results indicated that oxidation index had a median of 0.36 (ranging from 0.05 to 1.45) [19].

### Histological analysis

Only two devices were received with tissue samples for analysis. Both samples showed evidence of low inflammation, and neither had evidence of polyethylene wear debris. However, metallic debris was observed in both cases, though features of adverse local tissue reactions to debris from the articular CoCr surface were not observed in either case.

## Discussion

The present study combines quantitative and qualitative observations from the retrieval analysis of the PCM cervical disc replacement to identify potential causes of failure. Twenty-five retrieved PCM devices were analyzed for in vivo damage, wear, material loss, and other visible signs of damage. Visual assessments indicated light in vivo damage to the articular surfaces. High-resolution microscopy provided quantitative data on anchor heights, surface roughness, and line roughness, showing no significant differences between the retrieved devices and the exemplar as-manufactured device. Overall, most devices in this study were removed due to radiographic evidence of migration. Of the 25 devices included in the analysis, 22 were found to be loose or not well-fixed at the time of removal. Specifically, these devices showed no bony ongrowth, no damage from removal, or were reported to have migrated. These observations suggest that the devices were either grossly loose or lacked bony ongrowth on features designed to promote osseointegration. Consequently, only three devices showed evidence of potential fixation [17,[20], [21], [22], [23]].

In the present study, none of the devices showed migration of only the superior component, with the majority having only migration of the inferior component. Additionally, of these migrated inferior components, 11 of the devices were measured to have a focalized deviation in the corner of the posterior quadrant, toward the lateral left quadrant. This may be consistent with the reported impact of device placement on increased stresses. Devices with a ball-and-socket articulation may require more precise device implantation to avoid posterior stresses both on the facet joints and, in the case of the PCM, on the posterior quadrant of the device. This could indicate that the surgical placement of these devices is an important factor in long-term device success [24,25].

The metrology findings from the present study were inconsistent with the reported damage pattern by Postak et al., who concluded that the majority of wear debris and surface polishing were present on the anterior edge of both retrieved and simulated devices. However, the authors assessed an older generation of the PCM device than the version included in the present study. Additionally, these authors did not report clinical reasons for removal or present modes of failure, so this deviation may not be present in successfully fixed devices [26]. This correlation suggests increased stress or mechanical strain in the posterior quadrant aligns with the pattern of anterior migration. Therefore, the PCM design, consisting of two separate components with a large ball diameter intended to enable translational motion consistent with the natural motion of the cervical spine, may contribute to this increase in posterior stress [1].

Notably, the Mobi-C, another ball-and-socket CDR design, has very few reported cases of migration, and early to midterm follow-up studies have reported positive outcome without any no cases of device migration [8,[27], [28], [29], [30], [31]]. Additionally, only one case report presented migration of the ProDisc-C, another ball-and-socket device, in the current literature [[32], [33], [34], [35]]. However, both the PBackspaceMobi-C and the Prodisc-C have smaller diameter articulating balls compared to the PCM. This association between the increasing level of constraint and ball diameter affecting clinical success has been previously identified and should be further explored [36].

Conflicting literature is available regarding the prevalence of migration in CDRs. Prospective IDE and clinical trials have reported up to 7-year outcome of the PCM cervical device, with less than 4% of all devices failing due to migration. Additionally, biomechanical studies have identified the PCM device being stable in comparison to other devices under pullout testing conditions [37]. On the other hand, cross-sectional analyses of registry data have identified the PCM cervical disc as having the highest prevalence of migration, with failures attributed to migration being 84% to 94% of all failures [7,8]. This in comparison to all other ball-and-socket designs, which range from 21% to 60% [7,8]. However, the true clinical incidence of migration is unknown, as it is unclear how many devices have been implanted, removed, and unreported, or remain functional and in use. Additionally, these databases have tended to be conservative estimates, indicating that this may be an under-reporting of the complications, and the true complaint rates may be larger. However, of the PCM devices received at our institution, the majority had clearly been removed due to migration, indicating that this may be a significant complication that is being overlooked in clinical literature.

The present study determined that polyethylene wear debris was not likely to have contributed to the failure of the explanted devices. Additionally, there were no radiographic observations of osteolysis. This is consistent with previous simulator studies, which have suggested that cervical devices perform well in the body, producing minimal wear debris from the metal-on-polyethylene surfaces [3,26,38]. Although limited tissue samples were available in this study, metallic particles were detected, with no evidence of polyethylene wear debris in the available tissues. Additionally, metrology results showed that only nine devices had deviations exceeding the manufacturer’s tolerance for the polyethylene radius. Notably, only 7 devices had deviations over 30 µm, the reported tolerance, and of these, only 5 had deviations over 60 µm. Moreover, these devices had no signs of in vivo damage, suggesting that retrieval damage and handling may have contributed to these deviations, not in vivo wear. Further, oxidation analysis indicated that excessive polyethylene oxidation was not a factor, likely due to the limited time in vivo (49–525 days).

This study was not without limitations. There was a lack of clinical data, including basic demographic and radiographic information received. Further, nine devices excluded from the analysis were received with no data; however, based on date of receipt and earliest possible date of implantation, none of these could not have exceeded 2 years of in vivo. Accordingly, the maximum period of implantation was less than 2 years for all devices. Longer follow-up may have identified additional modes of failure based on longer time in situ. For some devices, only intraoperative imaging was available at the time of removal, with no prerevision imaging submitted. This limits the ability to determine whether device damage was caused by the intended use of the device or occurred following anterior migration. As the mean time to revision was 121±115 days, or approximately 4 months, revision surgeries were likely scheduled soon after migration was noted. This underscores the need for comprehensive imaging and documentation. Image quality was an additional limitation as many received images were poor quality; however, gross migration was still apparent. Further, limited tissue samples were received for histopathology. This limited the extent to which wear debris and tissue reaction, or inflammation, could be associated with the failures.

## Conclusion

This study presents a comprehensive retrieval analysis of the PCM cervical disc replacement, utilizing both qualitative and quantitative techniques to assess the mode of failure. Collectively, the retrieval findings, including lack of extraction damage and bony ongrowth, with the radiographic imaging, indicated that the majority of devices were removed due to lack of fixation and subsequent migration. Notably, the measured focalized posterior deviation correlating with anterior slippage may be unique to the large ball-and-socket design of the PCM device and the substandard bone fixation design of the first-generation PCM implant.

## Declarations of competing interests

The authors declare that they have no known competing financial interests or personal relationships that could have appeared to influence the work reported in this article.
